# The Alleviating Effect of *Lagerstroemia indica* Flower Extract on Stretch Marks through Regulation of Mast Cells

**DOI:** 10.3390/molecules27041274

**Published:** 2022-02-14

**Authors:** Miji Yeom, Hyanggi Ji, Jongheon Shin, Eunae Cho, De-Hun Ryu, Deokhoon Park, Eunsun Jung

**Affiliations:** Biospectrum, Life Science Institute, Yongin-si 16827, Korea; biout@biospectrum.com (M.Y.); biocr@biospectrum.com (H.J.); biowa@biospectrum.com (J.S.); biozr@biospectrum.com (E.C.); biosc@biospectrum.com (D.-H.R.); pdh@biospectrum.com (D.P.)

**Keywords:** striae distensae, stretch marks, *Lagerstroemia indica*, cell adhesion, mast cell activation, ECM component, fibroblast

## Abstract

Striae distensae (SD) or stretch marks are common linear scars of atrophic skin with disintegrating extracellular matrix (ECM) structures. Although fibroblasts contribute to the construction of ECM structure in SD, some studies have reported that mast cell degranulation causes the disruption of ECM in early SD lesions. *Lagerstroemia indica* flower (LIF) has traditionally been used in India as a diuretic. However, little is known about the effect and molecular action of *Lagerstroemia indica* flower extract (LIFE) on alleviating SD. This study evaluated the effects of LIFE on mast cell degranulation and the synthesis of ECM components in fibroblasts. LIFE inhibits the adhesion of rat basophilic leukemia (RBL) cells, RBL-2H3 on fibronectin (FN) and the expression of integrin, a receptor for FN, thereby reducing focal adhesion kinase (FAK) phosphorylation. In addition, LIFE attenuated the allergen-induced granules and cytokine interleukin 3 (IL-3) through the adhesion with FN. Moreover, the conditioned medium (CM) of activated mast cells decreases the synthesis of ECM components, and LIFE restores the abnormal expressions induced by activated mast cells. These results demonstrate that LIFE suppresses FN-induced mast cell activation and promotes the synthesis of ECM components in fibroblast, which indicates that LIFE may be a useful cosmetic agent for SD treatment.

## 1. Introduction

Striae distensae (SD) or stretch marks are common linear scars of skin. SD rarely causes major pathological problems, but from a cosmetic point of view, it imposes a psychological burden [1,2]. Most SD occurs in pregnant, adolescent, and obese women. The risk factors of SD are genetic factors, hormonal excess, and mechanical stress, but the hypotheses often conflict [3,4].

SD develops pigmentation over time: the initial red linear lesions are striae rubrae (SR), while the chronic pale lesions are striae albae (SA). Histological alteration of SR shows prominent features: increase of dermal oedema between melanocytes and keratinocytes and melanogenesis in epidermis, the cleavage of collagen and the reduction or fragmentation of FN, elastin (ELN), and fibrillin (FBN), which is cross-linking, in the reticular dermis. Thinned epidermis is shown with a flattening of the rete pegs [1,5,6]. The reorganization of collagen, FN, ELN, and FBN are thought to be a key cause in SD pathogenesis, and these genes are used as genetic markers for SD [7,8].

In early stretch marks, mast cell degranulation and macrophage activation are also observed in the reticular dermis, and elastolysis of extracellular matrix (ECM) is promoted [9]. Obese woman with SD revealed the increase of mast cells around the vessels in the dermis, and fragmented and thinned ELN fibers [10]. Protease and cytokines referred to as mast cell mediators are a necessary source for the breakdown of FBN and ELN, as well as the activation of metalloproteinase [11,12,13]. These implicate that mast cell mediators including elastases be proposed as a key initiatory step in SD. However, mast cell activation on SD pathogenesis has not yet been fully studied.

Mast cells can respond to environmental, biochemical, physical, and mechanical stimuli. A general study of mast cell activation has focused on the aggregation of the high-affinity Fc epsilonR1 alpha (FceR1) receptor by antigen-binding immunoglobulin E (IgE) as a chemical stimulus [14]. Other studies have revealed that physical and mechanical stimuli also induce the activation of mast cell [15,16,17]. Bindings of cell surface receptors to adjacent ECMs function as physical and mechanical stimuli, and promote mast cell activation [18,19]. Integrin is a cell surface receptor composed of heterodimeric transmembrane glycoproteins containing α and β subunits that bind to ECM proteins, such as FN, vitronectin, and fibrinogen containing an arginine-glycine-aspartic acid (RGD) sequence or connecting segment (CS)-1 peptide. In addition to functioning as mechanical anchors, integrin mediates the transmission of physical and mechanical stimuli into biochemical signals, which promotes the release of granules, protease, and inflammatory cytokines, leading to disruption of the ECM structure [20,21,22,23,24,25]. Physical and mechanical stimulation is one of the triggers for stretch marks. SD is caused by the rapid tissue expansion of weakened skin connective tissue [26,27]. Based on the evidence, we suggest that mast cell degranulation through the adhesion of integrin to FN could be a novel target for the treatment of SD. We first screened for substances that alleviate mast cell adhesion to FN and found that LIFE significantly inhibited mast cell adhesion to FN among various extracts.

*Lagerstroemia indica* (LI), family *Lythraceae*, is well known as Indian Crape myrtle. It is native to the Indian subcontinent, but it is distributed in many countries, including those of Southeast Asia, China, Korea, and Japan, due to its ease of cultivation. According to traditional Indian medical treatment, bark, leaves and flowers of LI are used as diuretic and fever remedy [28]. In addition, LI displays various biological properties that include anti-inflammatory, antipyretic, analgesic, anti-diabetic, antioxidant, and hepatoprotective activity [29,30,31,32]. However, the effects of LI and its molecular mechanism on SD have not yet been fully understood.

The purpose of this study is to evaluate LIFE as an inhibitor of mast cell activation and an activator of ECM component in fibroblast. We investigated the inhibitory effect of LIFE on FN-induced mast cell activation. Also, we tested the changes of ECM components in fibroblasts by mediators from activated mast cells and the recovery effect of LIFE on these alterations.

## 2. Results

### 2.1. LIFE Inhibits Mast Cell Binding to FN

RBL-2H3 cells are of mucosal mast cell origin, and they are a prime model for investigating immediate hypersensitivity reactions [33,34]. In addition, RBL-2H3 cells spontaneously adhere to FN, vitronectin, and fibrinogen, leading to activation of mast cells [22,35]. Following previous studies, we observed FN-induced mast cell activation using RBL-2H3 cells.

**Figure 1:**
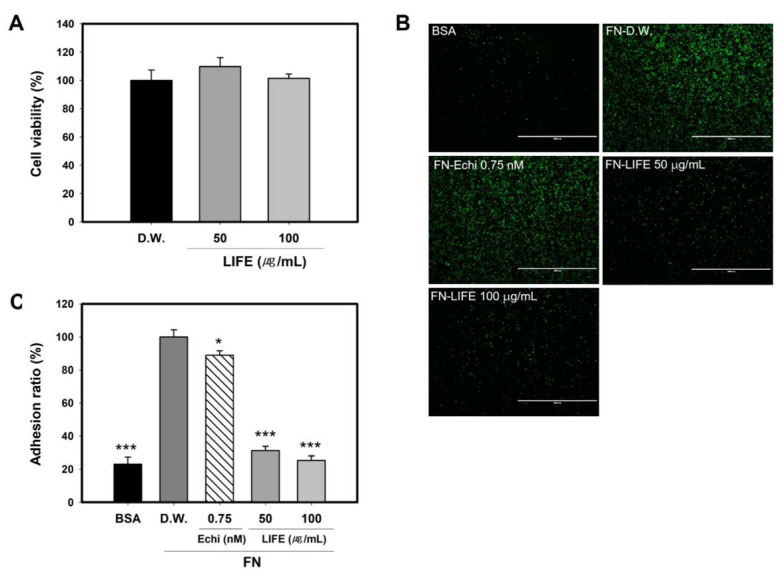
LIFE decreases the binding of rat mucosal mast cell line RBL-2H3 to FN. Cells (passage 3–7) at 90% confluence were pretreated with (50 or 100) μg/mL of LIFE for 24 h. (**A**) Cell viability of RBL-2H3 was measured by MTT assay. RBL-2H3 cells pretreated with LIFE were labelled by calcein-AM for 30 min at 37 °C, and then seeded onto FN-coated plates for 2 h. (**B**) Fluorescence intensity was observed under fluorescence microscopy, and was determined using Infinite M200. (**C**). Data are presented as mean ± SEM from three replicated measurements. One-way ANOVA followed by the post hoc Dunnett’s multiple comparisons test (*n* = 3, * *p* < 0.05, *** *p* < 0.001, compared with control-D.W. treated). Echi: echistatin, BSA: bovine serum albumin, FN-fibronectin, LIFE: *Lagerstroemia indica* flower extract.

Cytotoxicity of LIFE on a rat mucosal mast cell line, RBL-2H3, was measured by a MTT assay that measures cell metabolic activity in the cell culture. LIFE extracts did not show cytotoxicity to RBL-2H3 at 50 and 100 μg/mL of LIFE (Figure 1A). Under the non-toxicological levels, we investigated the effect of LIFE on mast cell adhesion.

As reported, RBL-2H3 cells adhered to FN without any stimuli, unlike its adherence to bovine serum albumin (BSA) (Figure 1B,C). To observe the effect of LIFE on the binding of mast cell to FN, we examined the adherence of RBL-2H3 pretreated by LIFE or echistatin (Echi), RGD inhibitor, as positive control, on the FN-coated plates. Echi hinders the interaction between integrin and ECM by competitively binding with integrin αvβ3, αⅡβ3, and α5β1 [36]. Figure 1C showed that the adhesion of RBL-2H3 to FN was significantly inhibited by (50 and 100) μg/mL of LIFE up to about (70 and 80) %, respectively. The adhesion of RBL-2H3 pretreated with Echi to FN was decreased by up to about 12% at 0.75 nM.These results suggest that LIFE could be an effective inhibitor for the adhesion of mast cell to FN.

### 2.2. LIFE Decreases the Expression of Integrin α_4_ and β_3_

Multiple integrin α3β1, α4β1, α5β1, and αvβ3 are involved in the adhesion to RGD sequence of FN. Among them, α4, α5, and β3 subunits play key roles in the adhesion to FN and the activation of RBL-2H3 cells [22].

**Figure 2:**
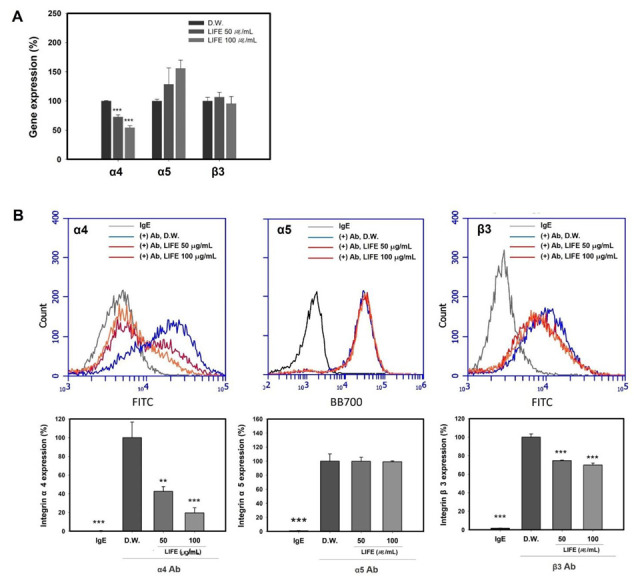
LIFE downregulates the expression of integrin α_4_ and β_3_ in RBL-2H3. Cells (passage 3–7) at 90% confluence were pretreated with (50 or 100) μg/mL of LIFE for 24 h. (**A**) Integrin α4, α5, and β3 mRNA were measured by quantitative real-time PCR. (**B**) Integrin α4, α5, and β3 on the surface of RBL-2H3 cells were measured by flow cytometry. Cells were harvested, and stained with antibodies for specific integrins on the surface of the cell. Data are presented as mean ± SEM from three replicated measurements. One-way ANOVA followed by the post hoc Dunnett’s multiple comparisons test (*n* = 3, ** *p* < 0.01, *** *p* < 0.001, compared with control-D.W. treated). Ab: Antibody, IgE: immunoglobulin E, LIFE: *Lagerstroemia indica* flower extract.

To reveal its mechanism of LIFE on the adhesion to FN, we measured the expression of integrin α4, α5, and β3 using cells pretreated with LIFE. Figure 2A shows that the expression of integrin α4 was decreased by LIFE at (50 and 100) μg/mL by ~(30 and 50) %, respectively, compared to control group. The expression of integrin α5 and β3 was not significantly altered by LIFE.

We next confirmed the number of integrins on the cell surface by using antibodies to integrins α4, α5, and β3 in flow cytometry. Figure 2B shows that LIFE at 100 μg/mL effectively reduced the number of integrin α4 by up to ~80%, consistent with the mRNA expression results. The expression of integrin β3 on cell surface was inhibited by LIFE at 100 μg/mL by up to ~30%. The expression of integrin α5 was not significantly changed by LIFE at 50 and 100 μg/mL. These results suggest that the inhibition of binding mast cell to FN by LIFE is regulated by the reduction of integrins α4 and β3, rather than α5, on the cell surface.

### 2.3. LIFE Inhibits FAK Phosphorylation but Not Intact FAK Protein

Integrin is a sensory molecule that converts mechanical information from the ECM into biochemical signals. The binding with FN promotes the clustering of integrins, which recruit signaling proteins to form a clustered adhesion complex, leading to the phosphorylation of signaling proteins like FAK [37,38,39]. From these references, we hypothesized that the reduction of integrins by LIFE could decrease the phosphorylation of FAK.

**Figure 3:**
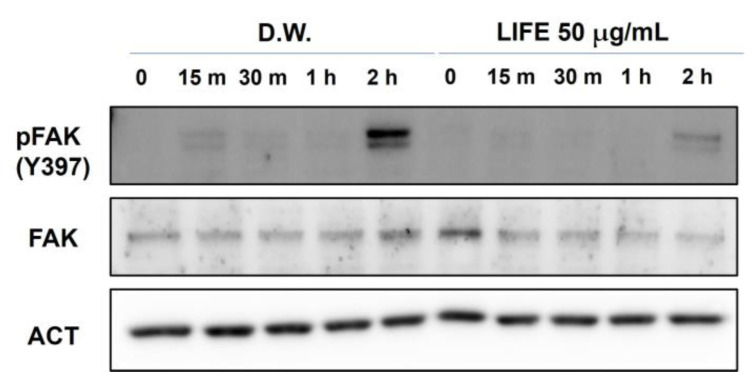
LIFE decreases FAK phosphorylation, but not intact FAK protein. Cells (passage 3–7) at 90% confluence were pretreated with 50 μg/mL of LIFE for 24 h. Cells were seeded onto FN-coated plates for 2 h, harvested at the indicated time, and then, analyzed by Western blot analysis with anti-Pfak(Y397), anti-FAK, and anti-Actin. FAK: focal adhesion kinase, ACT: actin, LIFE: *Lagerstroemia indica* flower extract.

Next, we tested whether LIFE changes the status of FAK phosphorylation. Following the procedure of the adhesion experiments (Figure 1B), RBL-2H3 cells pretreated with LIFE at 50 μg/mL for 24 h were seeded onto the FN-coated plates, and harvested every 30 min for up to 2 h. Figure 3 shows that the increase of phosphorylated FAK along with the adhesion time was inhibited by LIFE at 50 μg/mL, whereas the amount of FAK intact protein was not affected. The results suggest that the decrease of integrins by LIFE results in the inhibition of FAK phosphorylation, as well as the reduction of adhesion to FN.

### 2.4. LIFE Decrease the IgE-Induced Relases of Graunles and Cytokine IL-3 on FN-Coated Plate

Adhesion to the ECM synergistically promotes mast cell activation, along with FcεR1. Degranulation of RBL-2H3 in response to aggregation of the high-affinity FceR1 receptor by antigen-bound IgE was significantly promoted on FN-coated plates. IgE-induced mast cell degranulation on FN was reversible by RGD and CS-1 peptides, or by integrin antibodies [21,22,35]. This means that interaction between integrin and FN promotes IgE-induced mast cell degranulation.

**Figure 4:**
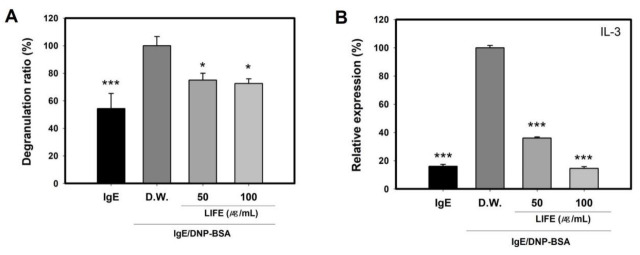
LIFE downregulates IgE-induced degranulation and release of cytokines from RBL-2H3 on FN-coated plate. Cells pretreated by (50 or 100) μg/mL LIFE for 24 h were sensitized by 1 µg/mL of IgE for 2 h, and then cells were seeded onto FN-coated plates with 25 ng/mL of DNP-BSA for 2 h. (**A**) The degranulation ratio was calculated by measuring the activity of β-hexosaminidase in supernatant and lysate. (**B**) IL-3 levels were measured by ELISA. Data are presented as mean ± SEM from three replicated measurements. One-way ANOVA followed by the post hoc Dunnett’s multiple comparisons test (*n* = 3, * *p* < 0.05, *** *p* < 0.001, compared with control-D.W. treated group). IgE: immunoglobulin E, DNP-BSA: 2,4-dinitrophenylated BSA.

To determine the inhibitory effect of LIFE on the IgE-induced degranulation on FN, we measured the degranulation ratio using the activated mast cells on FN-coated plate. For activation, RBL-2H3 cells pretreated with LIFE for 24 h were sensitized with IgE for 2 h, and then adhered to FN while triggering the cells with 2,4-dinitrophenylated BSA (DNP-BSA). After stimulation and adhesion for 2 h, the degranulation ratio was measured by the activity of β-hexosaminidase, a granule enzyme. Figure 4A shows that the degranulation ratio was inhibited by LIFE at 100 μg/mL by up to ~30%.

In addition, we examined the inhibitory effect of LIFE on the release of IL-3 known as inflammatory cytokines to response to FN [20]. IL-3 levels in supernatants from 24 h stimulated mast cells were analyzed using enzyme-linked immunosorbent assay (ELISA). Figure 4B shows that LIFE significantly inhibited the release of IL-3 at 100 μg/mL by up to ~86%. These results demonstrate that LIFE decreased IgE-induced degranulation and cytokine IL-3 on an FN-coated plate through the inhibition of the adhesion to FN.

### 2.5. Conditioned Medium from Activated RBL-2H3 Changes the Expression of ECM Components and Inflammatory Mediators in Fibroblast

In the early SD, mast cell activation in the mid dermis promotes the destruction of the ECM component. However, the relationship between mast cell activation and dermal fibroblasts in the regulation of ECM component known as genetic markers for SD has been little studied. We observed whether mediators released from activated mast cells can influence the synthesis of ECM components in fibroblasts.

**Figure 5:**
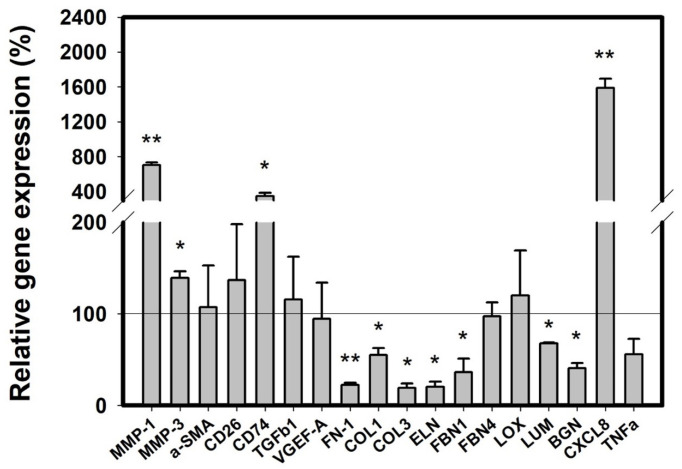
Conditioned medium from RBL-2H3 increases the expression of ECM components and inflammation mediator in NHDF. RBL-2H3 cells (passage 3–7) at 90% confluence was sensitized with 200 ng/mL of IgE for 24 h. Then, cells were activated with 100 ng/mL of DNP-BSA for 2 h. CM was collected from activated RBL-2H3 cells, and 50% CM or DMEM were treated onto NHDF for 24 h. The gene expression was measured by quantitative real-time PCR. Data are shown as relative value compared with CM non-treated control (line). Data are presented as mean ± SEM from three replicated measurements. Student’s *t*-test (*n* = 3, * *p* < 0.05, ** *p* < 0.01, compared with CM non-treated control).

We first examined the effect on normal human dermal fibroblasts (NHDFs) of treating conditioned medium (CM) from RBL-2H3 in response to antigen-bound IgE. A previous report found that the genes related to the decomposition of ECM and pro-inflammation were changed in the HDF isolated from a SD lesion [8]. Figure 5 shows that consistent with this, we found that most ECM components were decreased in the NHDF treated with the CM from RBL-2H3 in response to antigen-bound IgE, when compared to non-treated NHDF. The collagens type I (COL1) and III (COL3) were decreased by (45 and 80) %, respectively. FN and ELN were also reduced by (78 and 80) %, respectively. Also, biglycan (BGN) and lumican (LUM), which are necessary for the assembly of collagen fibrils, were decreased by (59 and 33) %, respectively. In our condition, lysyl oxidase (LOX) and FBN4 were not changed, although FBN1 was decreased by 64%. In the same condition, we found a marked difference on matrix metallopeptidase 1(MMP1). Its expression was strongly increased by 607%, while a mild increase was observed on the expression of MMP3 (37% of control). In addition, we found a significant increase of an inflammatory mediator, C-X-C motif chemokine ligand 8 (CXCL8) (1400% of control), but not tumor necrosis factor-α (TNF-α).

On the other hand, previous reports showed that cluster of differentiation 26 (CD26), a known marker of fibrogenic fibroblast, is upregulated, while in contrast, cluster of differentiation 74 (CD74), a known anti-fibrotic surface receptor, is downregulated in early SD lesion [40]. Also, the increase of alpha-smooth muscle actin (α-SMA) is thought to be a hallmark of early SD, distinguishing from late SD [27]. Unlike previous reports, the CM of mast cells in our condition did not cause any differences in α-SMA and CD-26 expression, but increased the expression of CD74. In agreement, transforming growth factor-β (TGF-β1), an inducer of collagen synthesis, and vascular endothelial growth factor A (VEGF-A), a stimulator of collagen deposition, showed no significant differences between treatment and non-treatment with CM from RBL-2H3 cells.

These results demonstrated that CM from RBL-2H3 in response to IgE increases the expression of ECM components and inflammatory mediator, like that shown in HDF isolated from an SD lesion.

### 2.6. LIFE Restores Abnormal Genes Expression in Fibroblast in Response to the Conditioned Medium from the Activated Mast Cells

**Figure 6:**
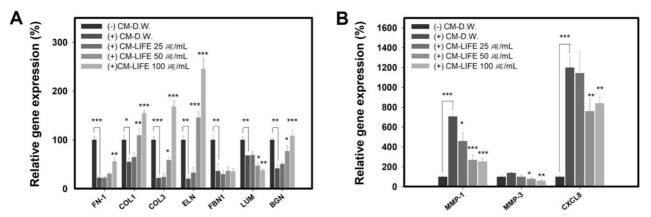
LIFE restores abnormal genes expression in NHDF in response to the conditioned medium from the activated RBL-2H3. CM of activated RBL-2H3 cells was obtained under the same conditions as in Figure 6. Fifty percent CM or DMEM was treated with (25, 50, or 100) μg/mL LIFE in NHDF. (**A**,**B**) The expression of genes was measured by quantitative real-time PCR. Data are shown as relative value compared with CM non-treated control. Data are presented as mean ± SEM from three replicated measurements One-way ANOVA followed by the post hoc Dunnett’s multiple comparisons test (*n* = 3, * *p* < 0.05, ** *p* < 0.01 *** *p* < 0.001, compared with (+) CM-D.W. treated group).

Based on these results, we next wondered if LIFE could restore abnormal expressions of genes involved in the decomposition of ECM and pro-inflammation induced by CM from RBL-2H3. As shown in Figure 6A, we observed that LIFE enhanced COL1, COL3, FN1, ELN, and BGN expression in dose-dependent manner, but not FBN1 and LUM. Also, MMP-1, MMP-3, and CXCL8 expressions were downregulated by LIFE at (25, 50, or 100) μg/mL in dose-dependent manner (Figure 6B). These results demonstrate that LIFE was essential for alleviating the abnormal expressions of genes involved in SD.

### 2.7. Phenolic Compounds Analysis of LIFE

**Figure 7:**
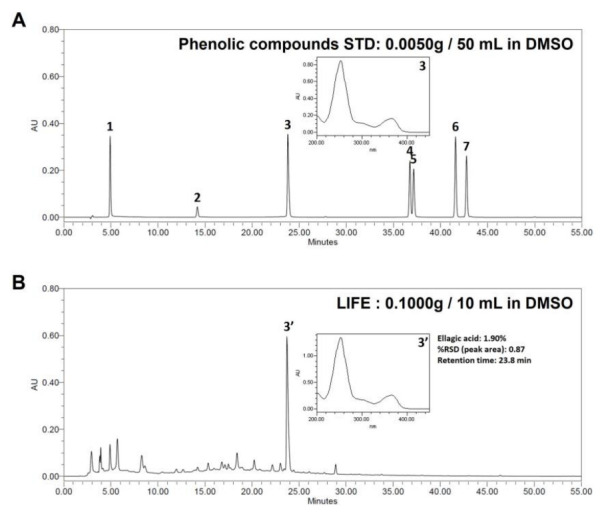
Composition of LIFE extracts. HPLC chromatogram of LIFE at 270 nm. (**A**) HPLC chromatogram of standard (STD) at 270 nm. 1—Gallic acid; 2—Catechin; 3—Ellagic acid; 4—Luteolin; 5—Quercetin; 6—Apigenin; 7—Kaempferol (**B**) HPLC chromatogram of LIFE at 270 nm. Contents values are presented as mean ± SEM from three replicated measurements. STD: standard, DMSO: dimethyl sulfoxide.

To identify the chemical composition of LIFE, we analyzed the phenolic compounds in LIFE by HPLC. The LIFE mainly contained 19.0 mg ellagic acid equivalent/g dried extract. Figure 7 shows that the major peak, tR of 23.8 min, was identified as ellagic acid. Its content was calculated as over (1.90 ± 0.87) % (*w*/*w*). Other phenolic compounds such as gallic acid, catechin, luteolin, quercetin, apigenin and kaempferol were not detected in LIFE.

### 2.8. Ellagic Acid Inhibits Mast Cell Binding to FN

**Figure 8:**
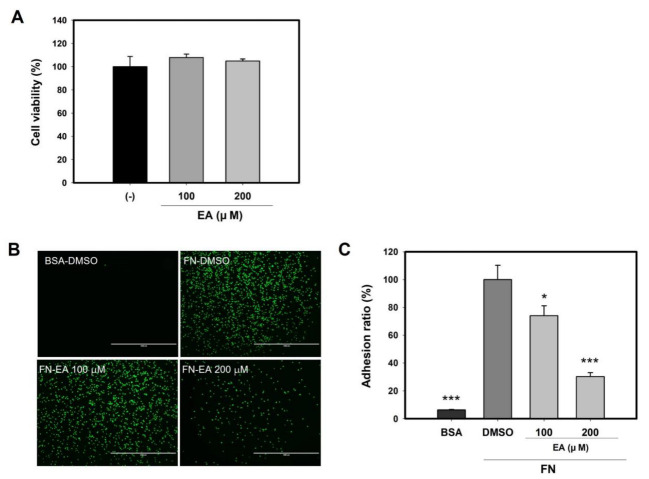
Ellagic acid inhibits the binding of RBL-2H3 to FN. Following the procedure in Figure 1, cells were pretreated with (100 or 200) μM ellagic acid for 24 h. (**A**) Cell viability of RBL-2H3 was measured by MTT assay. Cells labelled by calcein-AM were seeded onto FN-coated plates for 2 h. (**B**) The fluorescence intensity was observed under fluorescence microscopy, and (**C**) was determined using Infinite M200. Data are presented as mean ± SEM from three replicated measurements. One-way ANOVA followed by the post hoc Dunnett’s multiple comparisons test (*n* = 3, * *p* < 0.05, *** *p* < 0.001 compared with control-DMSO treated). EA: ellagic acid, BSA: bovine serum albumin, FN: fibronectin, DMSO: dimethyl sulfoxide.

To clarify the roles of ellagic acid, the inhibitory effect on mast cell adhesion was evaluated. Under the non-toxicological levels (Figure 8A), we tested the binding of RBL-2H3 cells pretreated with ellagic acid (EA) on FN. Figure 8C showed that the adhesion ratio of RBL-2H3 cells pretreated with ellagic acid to FN was decreased by up to ~70% at 200 μM. These results suggest that EA is one of the active ingredients contributing to the inhibitory effect on mast cell adhesion of LIFE (Figure 8B,C).

## 3. Discussion

LIFE inhibits the adhesion of RBL-2H3 on FN and decreases the expression of integrin, thereby leading to reduced FAK phosphorylation. Through the inhibition of the adhesion to FN, LIFE decreases IgE-induced degranulation on FN-coated plate. Although further experiments are needed, based on these results, we suggest that LIFE can be a novel agent for relieving stretch marks.

Mechanical stretching during rapid tissue expansion is one cause of the SD pathogenesis, owing to the perpendicularity of SD to the direction of skin [26]. Skin reveals various physiological and cellular responses to mechanical stress. Mechanical stimuli can be converted to biochemical responses through various cellular molecules, which include mechanosensitive ion channels, G-protein coupled receptors, protein kinases, integrinmatrix interactions, and other membrane-associated signal-transduction molecules [41]. Especially, it is well-known that interaction between integrin and ECM senses mechanical stress, and then activates intracellular pathway [42,43]. In the case of mast cell, mechanical stretching induces mast cell degranulation by the adhesion of integrin and FN [23]. These facts can provide an infinite link between integrin-FN interaction and mast cell degranulation, which is known as an early event in the pathogenesis of SD. Although further genetic experiments are needed, one possible pathogenic mechanism of SD is apparent: mechanical stress during tissue expansion induces mast cell degranulation through the adhesion of integrins to ECM, and subsequently promotes elastolysis. Based on this hypothesis, we suggest that the integrin-FN interaction-induced mast cell degranulation can be a new in vitro evaluation to screen for agents alleviating SD.

Some reports suggest that CD26 and α-SMA, known as fibrotic markers, increase in early lesions of SD [27,40]. However, we observed that CM of mast cells did not influence the expression of α-SMA and CD-26, but slightly increased the expression of CD74 known as an anti-fibrotic marker in fibroblast (Figure 6). In the relationship between mast cells and fibroblasts, many studies have addressed the pro-fibrotic or anti-fibrotic role of MCs in models of fibrosis, with partially conflicting results. Because this seems particularly relevant to the degree of stimuli given, results can change, depending on the experimental design [44]. From the aspect of the anti-fibrotic role, evidence is also prominent; extensive elastosis in sun-exposed skin was related to the increased mast cell prevalence or CM of mast cells, which reduces collagen and provokes pro-inflammatory signal in human tenocytes [45,46]. Despite controversy over the role of mast cells, our data showed that mediators from activated mast cells increases the expression of ECM components and inflammatory cytokine, like that shown in HDF isolated from an SD lesion. Therefore, we suggest that in vitro evaluation using the CM obtained from activated mast cells will be a good tool to screen for cosmetic agents for the treatment of SD.

In our study, we found that LIFE contains a higher proportion of EA (Figure 7). EA is a natural polyphenolic compound with strong antioxidant and anti-proliferative properties that is found in many fruits, seeds, and vegetables. The results from recent research have shown that EA has anti-proliferative, anti-atherogenic, anti-inflammatory, neuroprotective, and anti-carcinogenic effects. In anti-inflammatory activity, EA inhibits the binding and recruitment of circulating monocytes to vascular endothelial cells by decreasing the expression of adhesion molecules [47,48]. In addition, EA attenuates IgE-mediated allergic response in mast cells [49]. In the present study, EA inhibits the adhesion of mast cells to FN. There is the possibility that ellagic acid is an active compound of LIFE for inhibitory effect on FN-induced mast cell activation, although further study is needed.

In order to become an inhibitory active compound in LIFE, EA needs to show efficacy at a concentration of 0.95 μg/mL (1.9% in extract). However, we observed that EA showed inhibitory efficacy at a concentration of 100 μM (≈ 30 μg/mL) (Figure 8). Previous studies have shown that, in addition to EA, LI also contains various phenolic compounds, including 3-*O*-methyl gallate, isovitexin, vitexin, orientin, pyrogallol, and vanillic acid [29,31]. Therefore, we suggest that there are other active compounds with superior inhibitory activity than EA or show synergistic effects with EA. To identify the active compound inhibitory activity on mast cell adhesion, we are currently in the process of bioactivity-guided fractionation.

## 4. Materials and Method

### 4.1. Preparation of LIF Extracts

LIFE was obtained from the Jeonglim Agricultural Association (Namwon, South Korea). Water extract of LIFE was prepared by reflux extraction in purified water at (90–95) °C for 3 h. The extracts were filtered through filter paper. After spray drying, a perfectly dried LIFE was obtained. The obtained extract was dissolved in distilled water for further experiments.

### 4.2. Cell Culture and Cell Treatment

Rat mucosal mast cell line (RBL-2H3; CRL-2256^™^, ATCC, Manassas, VA, USA) was maintained in ATCC-formulated Eagle’s minimum essential medium (30-2003, ATCC) supplemented with 15% fetal calf serum and 1% penicillin/streptomycin (Gibco). Normal human dermal fibroblast (NHDF; PCS-201-012^™^, ATCC, Manassas, VA, USA) were maintained in Dulbecco’s modified Eagle’s medium (Welgene, Daegu, Korea) supplemented with 10% fetal calf serum and 1% penicillin/streptomycin (Gibco). Cells were maintained at 37 °C, under 5% CO_2_.

For treatment with LIFE, echistatin (3202, R&D system), and ellagic acid (5070, ChromaDex^™^), RBL-2H3 cells (passage 3–7) at 90% confluence were treated with substances for 24 h.

### 4.3. Adhesion Assay

The method was based on Lam et al., and modified [50]. RBL-2H3 cells were washed with 0.1% BSA + EMEM, resuspended at 1 × 10^6^ cells/mL, and labeled with 3 μg/mL of Calcein-AM (17783, Merck, Darmstadt, Germany) for 30 min at 37 °C. After labeling, cells were washed three times, and resuspended at 1 × 10^6^ cells/mL in 0.1% BSA + EMEM. The 100 μL of cell mixture was seeded onto an FN-coated 96-well plate (CWP001, R&D systems) and incubated at 37 °C. After 1 h, the fluorescence of total cells was measured with Infinite M200, washed with 0.1% BSA + EMEM three times, and then the remaining cells were measured. The degree of adhesion is expressed as the percentage of fluorescence remaining in the wells, after washing away unbound cells.

### 4.4. β-Hexosaminidase Release Assay

The method was based on John et al., and modified [24,35]. RBL-2H3 cells were resuspended in complete EMEM medium at a concentration of 3 × 10^6^ cells/mL, and were incubated with 1 μg/mL of IgE-DNP (D8406, Sigma) for 2 h at 37 °C. The cells were then washed three times, and resuspended in HBSS containing 1.5 mM of CaCl_2_ and 0.2% BSA. Next, cells were seeded to FN-coated 24-well plates, and allowed to adhere with 25 ng/mL DNP-BSA (324101, Sigma) for 2 h. The FN-coated 24-well plates were made as reference. The 24-well non-treated plates (32024, SPL) were coated with 50 μg/mL human FN (f1141, Sigma) in phosphate-buffered saline (PBS) for 16 h at 4 °C, washed three times with PBS, blocked with 3 % bovine serum albumin (BSA; a9418, Sigma) in HBSS (Hanks balanced salt solution-modified; 14025-092, Gibco) for 1 h at 37 °C, then washed 3 times with PBS. The degranulation ratio of the activity of β-hexosaminidase in the culture supernatants was measured.

### 4.5. Enzyme-Linked Immunosorbent Assay

Cells were stimulated with IgE-DNP and DNP-BSA, following the method of the β-hexosaminidase release assay. For ELISA assay, cells were allowed to adhere for 24 h. Supernatant obtained at the indicated time was analyzed by IL-3 (ab277709, abcam) ELISA kit, following the manufacturer’s instructions.

### 4.6. RNA Isolation and Quantitative Real-Time RT–PCR

Total RNA was extracted using the Qiagen RNeasy Mini Kit (Qiagen, Hilden, Germany), following the manufacturer’s instructions. Complementary DNA was obtained by the reverse transcription of 1 μg of total mRNA using amfiRivert cDNA synthesis platinum master mix (GenDEPOT, Katy, TX, USA), following the manufacturer’s instructions. Real-time reverse transcription PCR was done using an ABI PRISM 7500 (Applied Biosystems, Waltham, MA, USA), following the manufacturer’s instructions. Table 1 lists the primer sequences. The primer sequences of all genes used in Figure 5 and Figure 6 followed the method of Perez-Aso et al. [8].

### 4.7. Flow Cytometry Analysis

Cells were incubated with FITC-conjugated anti-CD49d(α4) (557457, BD) and anti-CD61(β3) (561909, BD) and BB700-conjugated anti-CD49e(α5) (745884, BD). FITC-conjugated mouse IgG2a (553456, BD), FITC-conjugated mouse IgG1 (550616, BD), and BB700 hamster IgG1 were used as negative control, respectively. The detailed method was followed in accordance with the manufacturer’s instructions. Cells were analyzed with an EVOS^®^ FL Cell Imaging System (Thermo Fisher Scientific, Waltham, MA, USA) and a BD Accuri^™^ C6 Plus (BD Biosciences, Franklin Lakes, NJ, USA).

### 4.8. Western Blotting

Cell lysates were prepared with protein extraction reagent (71009-3, Novagen) with protease inhibitor and phosphatase inhibitor (P3100, P3200, GenDEPOT). Total proteins were separated by a NuPAGE electrophoresis system (Thermo Fisher Scientific, Waltham, MA, USA) and transferred to polyvinylidene difluoride (PVDF) membranes. Immunoblotting was performed using primary antibody against pFAK(Y327) (3283, Cell Signaling Technology, Danvers, MA, USA), FAK (AF4467, R&D system) and β-actin (Santa Cruz Biotechnology, Inc., Dallas, TX, USA). Scanning densitometric values of bands were analyzed using ImageJ, software version 1.52a (National Institutes of Health, Bethesda, MD, USA).

### 4.9. Preparation of Activated Mast Cell-CM and Cell Treatment

RBL-2H3 cells were cultured until reaching 90% confluence. For stimulation, cells were incubated with 200 ng/mL of IgE in 2% FBS + EMEM, washed three times with PBS, and 100 ng/mL of DNP-BSA added in 2% FBS + EMEM for 2 h. The conditioned medium was collected, centrifuged at 10,000 rpm for 10 min, and filtered through a 0.22-µm syringe filter. Supernatants were collected and stored at −80 °C. The 50% CM or DMEM were treated on NHDF for 24 h.

### 4.10. High-Performance Liquid Chromatography (HPLC)

Identification and quantification of phenolic compounds in the LIFE were performed using Waters HPLC system (Waters, Milford, MA, USA) composed of Waters 2695 Separation module and Waters 996 PDA. A Phenomenex Luna C18 column (4.6 mm × 250 mm, ID 5 μm) was used for separation. The sample injection volume was 10 μL. The signal was monitored at 270 nm. Elution gradient: 10% organic phase B, hold for 5 min; from 10 to 30% organic phase B in 25 min (linear gradient); from 30 to 50% organic phase B in 20 min (linear gradient); from 50 to 100% organic phase B in 10 min (linear gradient); 100% organic phase B, hold for 10 min; then back to the starting condition in 1 min and re-equilibration for 9 min. The flow rate was 1.0 mL/min. Each analysis required 80 min, including the re-equilibration time.

### 4.11. Statistical Analysis

Data were presented as mean ± SEM from three replicated measurements and analyzed by Student’s *t*-test or one-way ANOVA followed by the post hoc Dunnett’s multiple comparisons test between two groups or among multiple groups. *p* < 0.05 was considered statistically significant.

## 5. Conclusions

LIFE inhibits FN-induced mast cell activation through the regulation of integrin expression. In addition, LIFE restores the alteration of ECM-related or inflammatory genes by mediators from activated mast cells. These results suggest that LIFE can be a novel natural plant source for relieving stretch marks.

## Figures and Tables

**Table 1:** List of primer sequences.

Gene Name	Accession Number	Primer Sequence
Integrin α4	NM_001107737.1	F: ctccccacaggcctttattt
		R: tctctgtcacgtcgcagttt
Integrin α5	NM_001108118.1	F: agctgcatttccgagtctg
		R: ctcacactgaaggctgaacg
Integrin β3	NM_153720.1	F: cacctgcatgtccaccaa
		R: cagctgccacactcacagtt

## Data Availability

Data will be made available on request.

## Sample Availability

Not available.

## List of Abbreviations

**Abbreviations**	**Full Name**
DNP-BSA	2,4-dinitrophenylated BSA
ACT	actin
α-SMA	alpha-smooth muscle actin
RGD	arginine-glycine-aspartic acid
BGN	biglycan
BSA	bovine serum albumin
CXCL8	C-X-C motif chemokine ligand 8
CD26	cluster of differentiation 26
CD74	cluster of differentiation 74
COL1	collagen type I
COL3	collagens type III
CM	conditioned medium
CS	connecting segment
DMEM	Dulbecco’s modified Eagle’s medium
EMEM	eagle’s minimum essential medium
Echi	echistatin
ELN	elastin
ELISA	enzyme-linked immunosorbent assay
ECM	extracellular matrix
FceR1	Fc epsilonR1 alpha (FceR1)
FBN	fibrillin
FN	fibronectin
FAK	focal adhesion kinase
HBSS	hanks balanced salt solution
IgE	immunoglobulin E
IL-3	interleukin-3
LI	*Lagerstroemia indica*
LIF	*Lagerstroemia indica* flower
LIFE	*Lagerstroemia indica* flower extract
LUM	lumican
LOX	lysyl oxidase
MMP1	matrix metallopeptidase 1
MMP3	matrix metallopeptidase 3
NHDF	normal human dermal fibroblast
PBS	phosphaste-buffered saline
RBL	rat basophilic leukemia
SA	striae albae
SD	striae distensae
SR	striae rubrae
TGF-β	transforming growth factor-β
TNF-α	tumor necrosis factor-α
VEGF-A	vascular endothelial growth factor A
